# 1197. Development and Implementation of Innovative Syringe Service Programs for Veterans in Rural and Urban Settings

**DOI:** 10.1093/ofid/ofab466.1389

**Published:** 2021-12-04

**Authors:** Minh Q Ho, Elizabeth Dinges, Karen Slazinski, Jacqueline Byrd, Mohammed Ahmed

**Affiliations:** 1 Orlando VA Healthcare System, 14014 Deep Forest Court, Florida; 2 Veteran Affairs, Springfield, Illinois; 3 Orlando VA HCS, Orlando, Florida; 4 Orlando VA Medical Center, Orlando, Florida

## Abstract

**Background:**

Syringe Services Programs (SSPs) is one aspect of a comprehensive Harm Reduction approach necessary to reduce the transmission of blood borne infections including Hepatitis B, Hepatitis C, and HIV. Substance Abuse and Mental Health Services Administration (SAMHSA) estimates that in 2019 that 595,000 veterans engage in opioid misuse with at least 57,000 veterans engaging in heroin. Stigmas to SSP are pervasive in the community and within the government system. Federal law prohibited the use of federal funds to purchase sterile needles or syringes for the purposes of illegal use of drugs by injection. It was officially clarified in May 24, 2021 that the prohibition to purchase syringes does not apply to Veterans Health Administration (VHA). While awaiting approximately 2 years to secure this clarification, syringes were obtained through a community donation. We aim to describe our process including difficulties encountered and data collected for SSP at two locations. Difficulties included developing an anonymous process to track quality, motivating providers to refer, educating highest risk veterans, providing face to face engagement during COVID-19 pandemic and ability to mail Harm Reduction kits containing sterile syringes.

Illiana VA Program Information Sheet

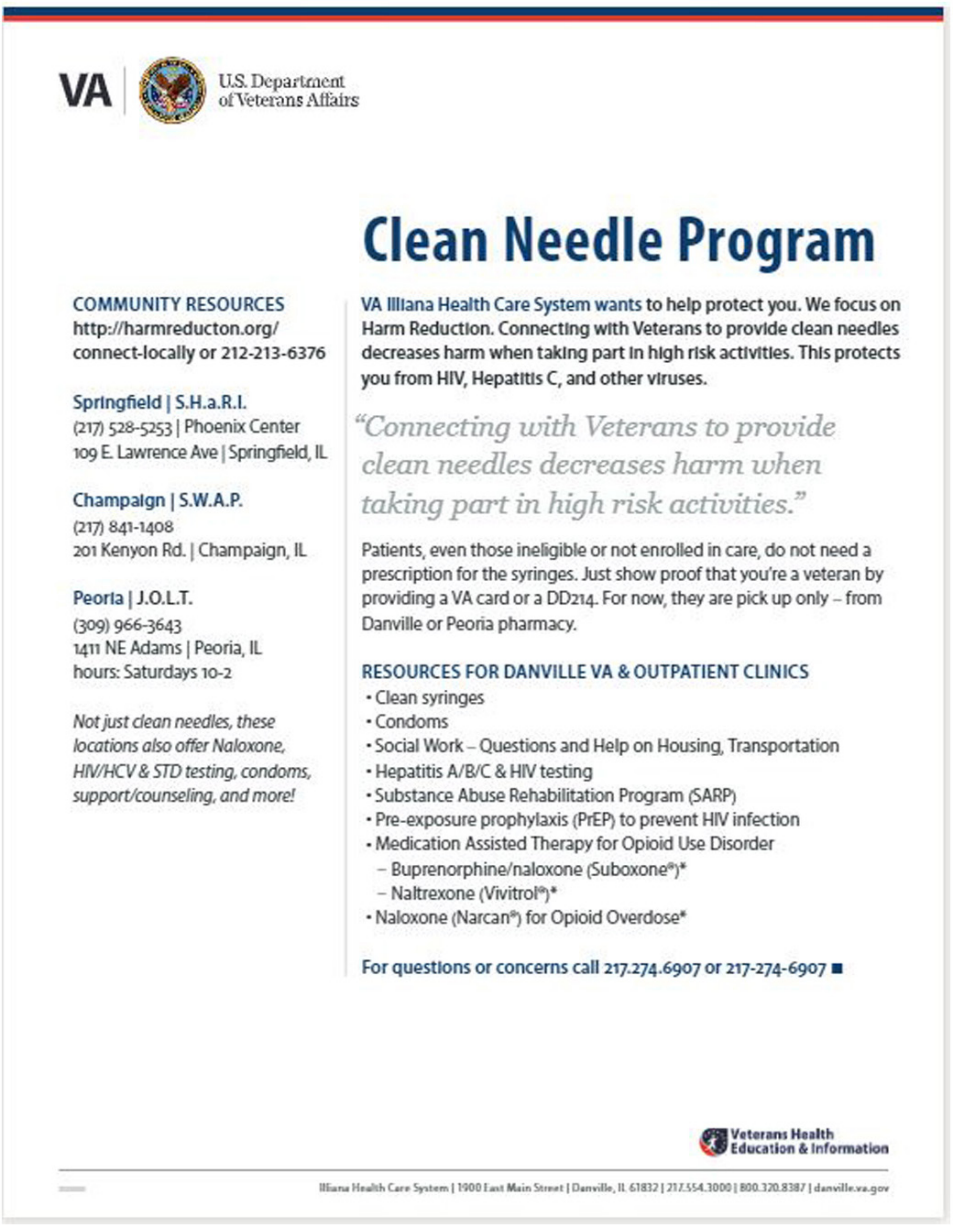

Orlando VA Program Information Sheet

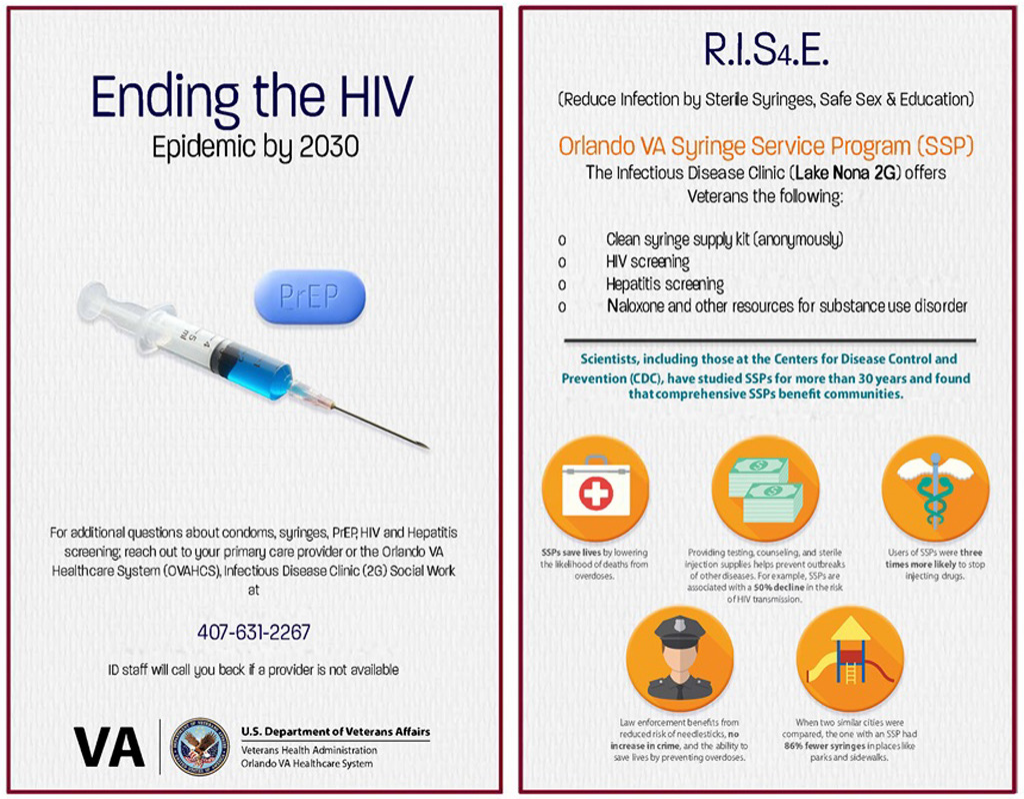

**Methods:**

Of the 140 facilities within VHA, there are currently only two SSPs established, Illiana VA and Orlando VA. A retrospective analysis of Harm Reduction benefits was performed among veterans who engaged with the two SSPs between 2018 to 2021.

Orlando VA SSP Intake Process Map

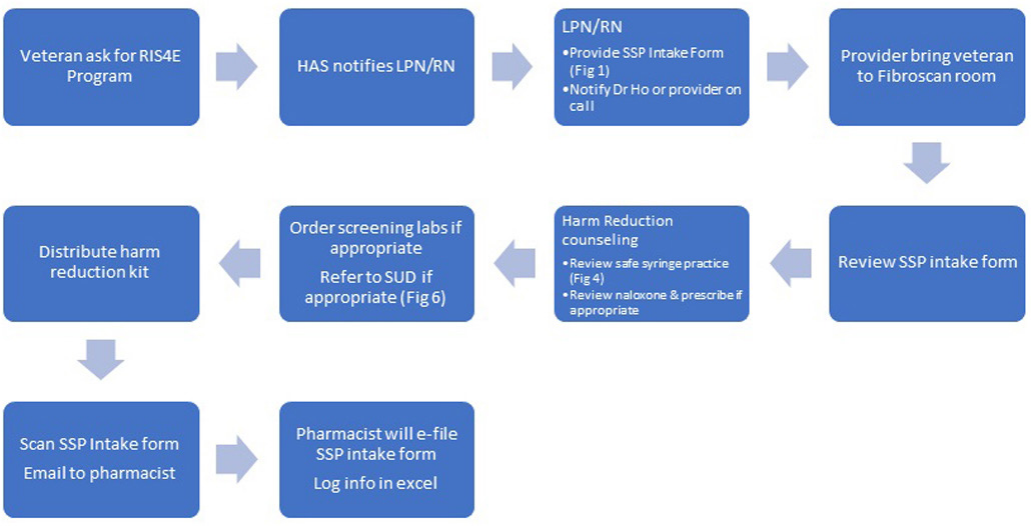

Process that veteran undergo when they engage with Orlando VA SSP

Contents of Standard SSP Kit Distributed to Veterans at Orlando VA

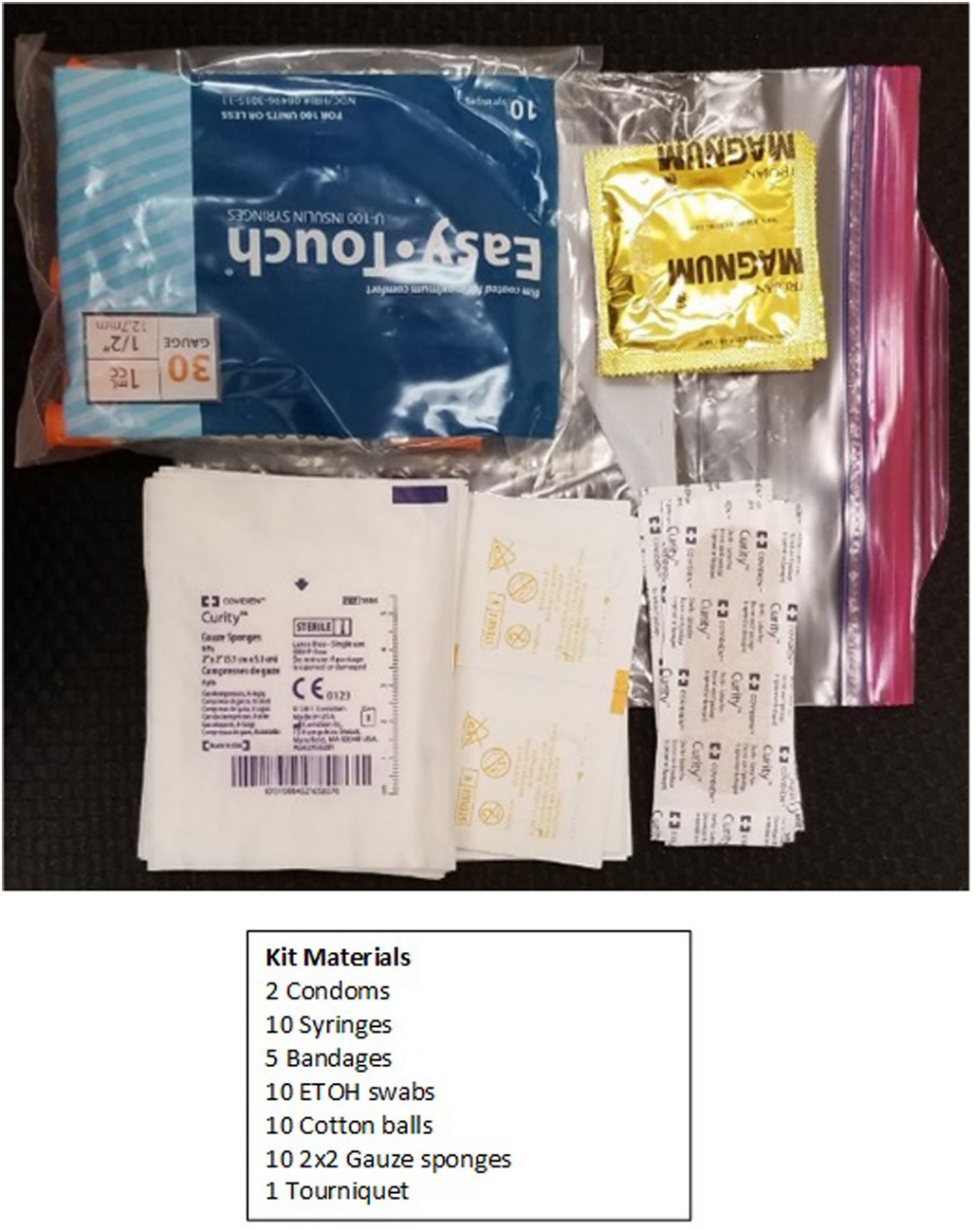

**Results:**

Approximately 3000 syringes were dispensed. Of the 17 veterans, 65% received syringes, 82% received naloxone, 100% engagement in mental health and 94% engagement in substance use disorder clinics. In total, 65% were screened for HIV, 82% for HCV and 29% for sexually transmitted infections.

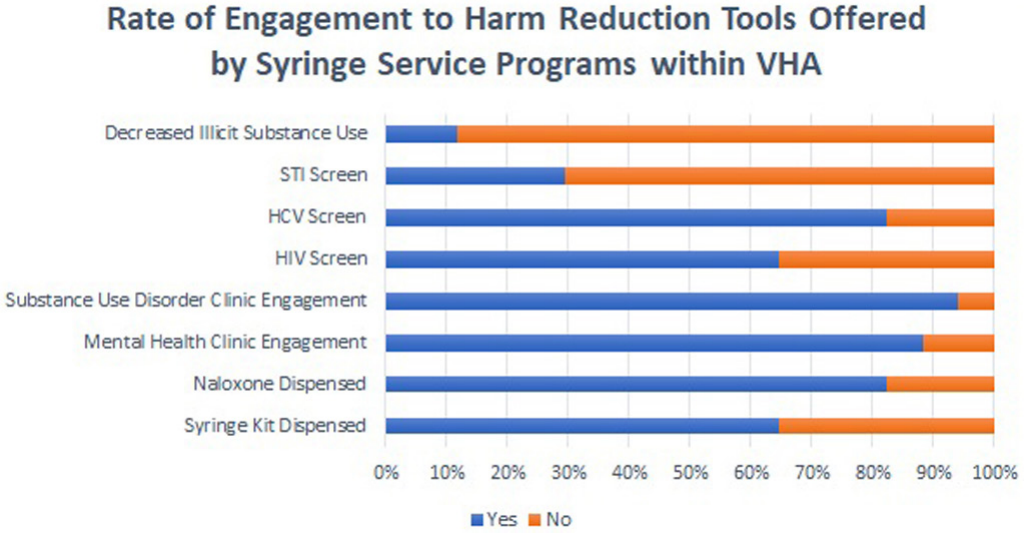

**Conclusion:**

These numbers, while modest, are notable, especially given the financial and organizational barriers that were in place. Furthermore, the COVID-19 pandemic impacted full implementation and outreach. With the recent, official clarification on syringe purchase and support for SSPs, the number of SSPs in the VA will grow, along with opportunity for more robust data collection. The experience of both facilities is a model for programs currently in development and moves us closer to ending the HIV epidemic by 2030.

**Disclosures:**

**All Authors**: No reported disclosures

